# Follicle-stimulating hormone-secreting pituitary adenoma manifesting as recurrent ovarian cysts in a young woman – latent risk of unidentified ovarian hyperstimulation: a case report

**DOI:** 10.1186/1756-0500-6-408

**Published:** 2013-10-11

**Authors:** Tomohiro Kawaguchi, Yoshikazu Ogawa, Kenji Ito, Mika Watanabe, Teiji Tominaga

**Affiliations:** 1 Department of Neurosurgery, Kohnan Hospital, 4-20-1 Nagamachi Minami, Taihaku-ku, Sendai, Miyagi 982-8523, Japan; 2 Department of Neurosurgery, Yonezawa City Hospital, Yonezawa, Yamagata, Japan; 3 Deparment of Pathology, Tohoku University Hospital, Sendai, Miyagi, Japan; 4 Deparment of Neurosurgery, Tohoku University Graduate School of Medicine, Sendai, Miyagi, Japan

**Keywords:** Follicle-stimulating hormone, Pituitary adenoma, Estradiol, Ovarian, Hyperstimulation syndrome

## Abstract

**Background:**

Ovarian hyperstimulation caused by follicle-stimulating hormone-secreting gonadotroph cell adenoma is a rare, with a few reported cases, but almost certainly unnoticed cases occur because of the absence of detailed examinations. We retrospectively reviewed 200 patients treated for gonadotroph cell adenoma in our institute and identified 26 women of reproductive age. Two of these 26 patients had a history of ovarian cysts. One patient was considered to have had typical ovarian hyperstimulation, successfully treated by transsphenoidal surgery. The other patient initially underwent transsphenoidal surgery because of visual disturbance, but endocrinological examinations suggested possible relationships with previous ovarian hyperstimulation. We present the former case and discuss the latent risk of failure to identify this entity.

**Case presentation:**

A 36-year-old woman with a sellar tumor was referred to our hospital with suspected ovarian hyperstimulation. She had a history of repeated surgery for ovarian cysts. Serum follicle-stimulating hormone and estradiol levels were within the normal ranges, and only the luteinizing hormone level was suppressed significantly. Transsphenoidal surgery achieved gross total tumor removal, and the histological diagnosis was follicle-stimulating hormone-secreting gonadotroph cell adenoma. The serum follicle-stimulating hormone, luteinizing hormone, and estradiol levels returned to the normal ranges postoperatively, and the ovarian cysts subsequently decreased in size without particular interventions.

**Conclusion:**

Ovarian hyperstimulation could regress after resolving the causes of high follicle-stimulating hormone level, so avoiding unnecessary ovary surgery. Detailed endocrinological examination including estradiol evaluation with pituitary imaging is quite important in women of reproductive age to establish the correct diagnosis.

## Background

Pituitary adenomas have been identified with increasing frequency in the last decade. Gonadotroph cell adenomas are the most common histological subtype, accounting for approximately 80% of non-functioning pituitary adenomas and 40% of all clinically recognized macroadenomas [1,2]. Immunohistochemical examination reveals gonadotropin production in adenoma cells, such as follicle-stimulating hormone (FSH), luteinizing hormone (LH), and/or a-subunit. However, secretion is usually low, so that these hormones frequently fail to manifest as clinical signs and symptoms [2]. Neurological symptoms occur only after the tumor has compressed the optic chiasm, resulting in visual disturbance. Therefore, gonadotroph cell adenomas are often diagnosed as non-functioning pituitary adenomas.

Ovarian cyst is a common disorder in young women with various etiologies. Ovarian hyperstimulation induced by inappropriate FSH oversecretion from adenoma cells may manifest as multiple ovarian cysts, although FSH-secreting gonadotroph cell adenoma is rare as the cause of multiple ovarian cysts [3]. A few patients with FSH-secreting gonadotroph cell adenomas have presented with symptoms of gonadotropin oversecretion [4-7]. The endocrinological profiles of these reported cases generally showed normal to slightly high FSH concentration and extremely low LH concentration, which are indicators for diagnosis [3]. However, the incidence of ovarian hyperstimulation caused by pituitary adenoma is unknown because of the absence of detailed gynecological examination.

We report a case of FSH-secreting pituitary adenoma associated with recurrent ovarian cysts, and describe the characteristics of gonadotroph cell adenoma in women of reproductive age to identify the incidence and clinical characteristics of this pathology.

## Case presentation

**Figure 1:**
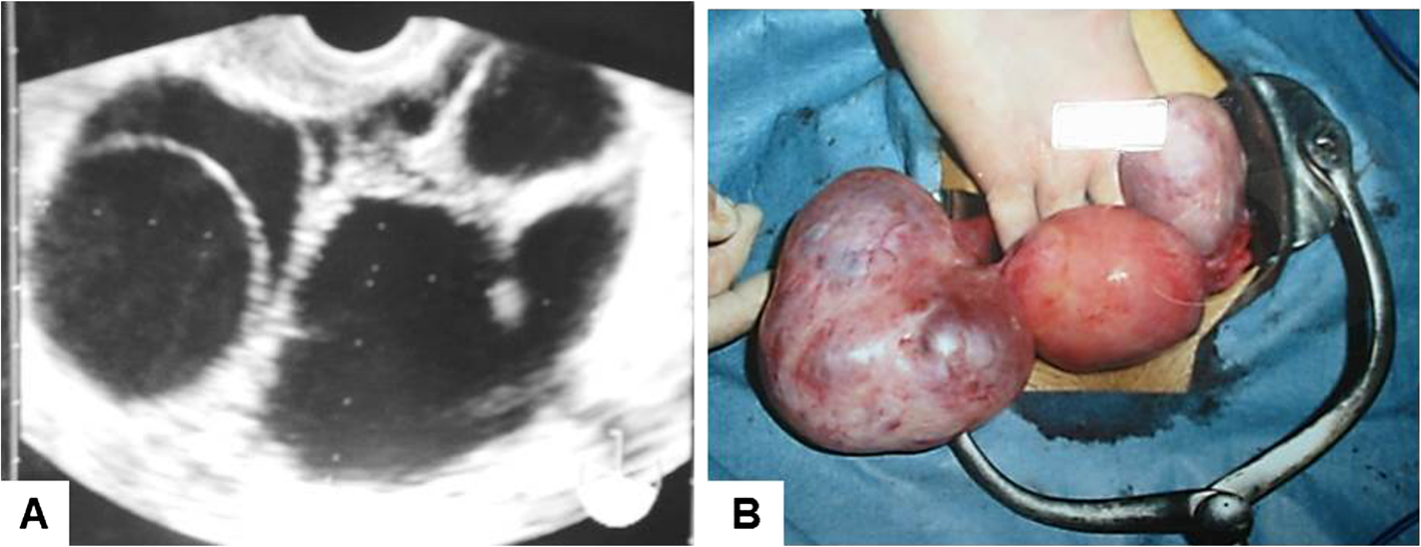
**Transvaginal ultrasonograms.** At initial surgery for ovarian cyst, the ovaries contained enlarged multiple cysts **(A)**. Photograph of the enlarged ovary with multiple cysts at surgery **(B)**.

**Figure 2:**
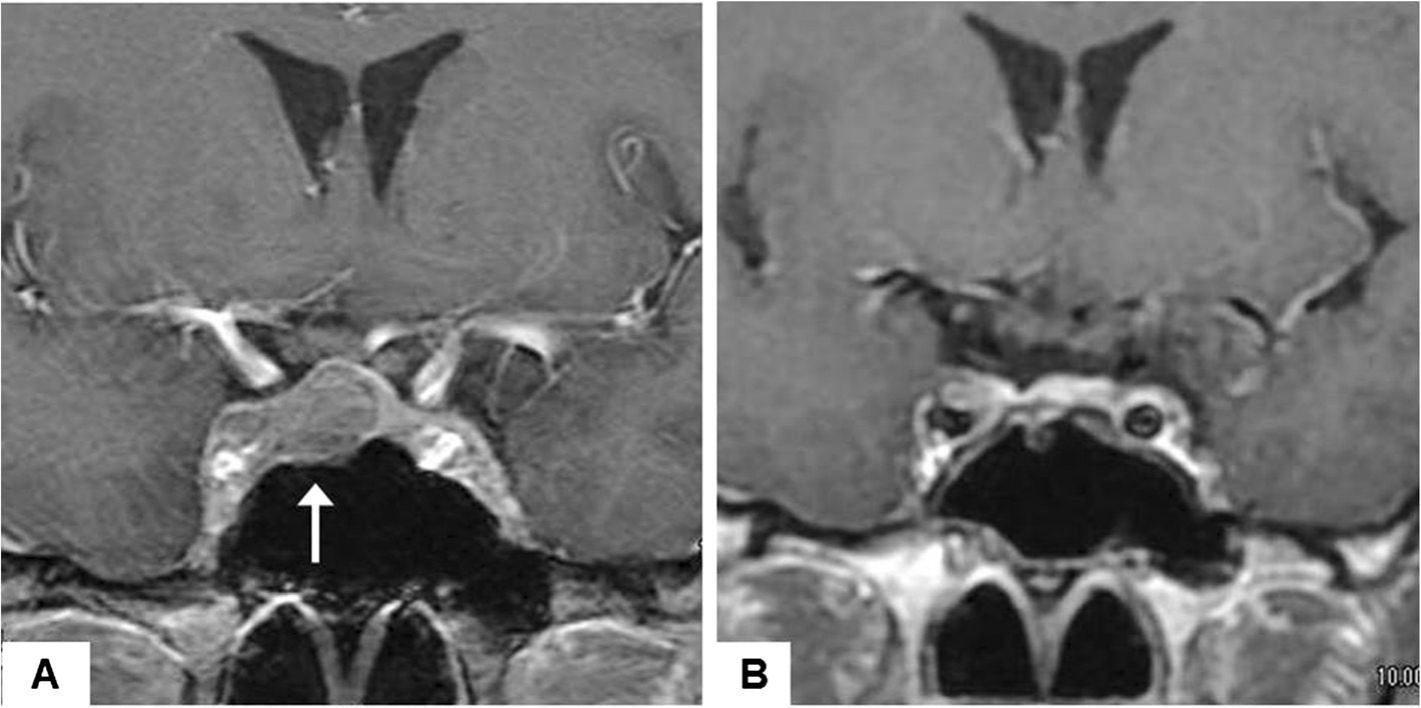
**Serial magnetic resonance images of the head.** Pretreatment coronal images showing a sellar-suprasellar mass lesion (**A**, arrow). Postoperative coronal images showing the tumor was grossly totally removed by a transsphenoidal approach **(B)**.

A 36-year-old woman with a sellar tumor was referred to our hospital. She had a history of repeated ovarian cysts. She presented with abdominal pain and metrorrhagia at age 32 years, and transvaginal ultrasonography revealed bilateral enlarged ovaries with multiple cysts (expanded follicles) (Figure 1A). No ascites was detected. The diagnosis was ovarian cyst and enucleation surgery was performed (Figure 1B). Her endocrinological profile after ovary surgery was FSH 12.0 mIU/ml (normal range for follicular phase, 3.01-14.72 mIU/ml) and estradiol 1820 pg/ml (normal range for follicular phase, 20–350 pg/ml). However, she still suffered metrorrhagia, so administration of oral contraceptives was started. Three years later, the ovarian cysts recurred, and her endocrinological profile was FSH 10.92 mIU/ml, LH <0.10 mIU/ml (normal range for follicular phase, 1.76-10.24 mIU/ml), estradiol 304 pg/ml, and prolactin 56.68 ng/ml (normal range, 4.91-29.32 ng/ml), which indicated that the serum FSH and estradiol levels were within the normal ranges, and serum LH level was significantly suppressed. Magnetic resonance imaging of the brain revealed a sellar mass lesion homogeneously enhanced by gadolinium, with diameters of 18 × 11 × 10 mm, which had compressed the optic chiasm upwards (Figure 2A). She was transferred to our hospital and transsphenoidal surgery achieved gross total tumor removal (Figure 2B). Postoperative course was uneventful, and serum concentrations of FSH, LH, and estradiol were 8.81 mIU/ml, 3.10 mIU/ml, and 99 pg/ml, respectively, all within the normal ranges. She was discharged 12 days after the surgery, and the ovarian cysts decreased in size without particular interventions, and did not recur again.

**Figure 3:**
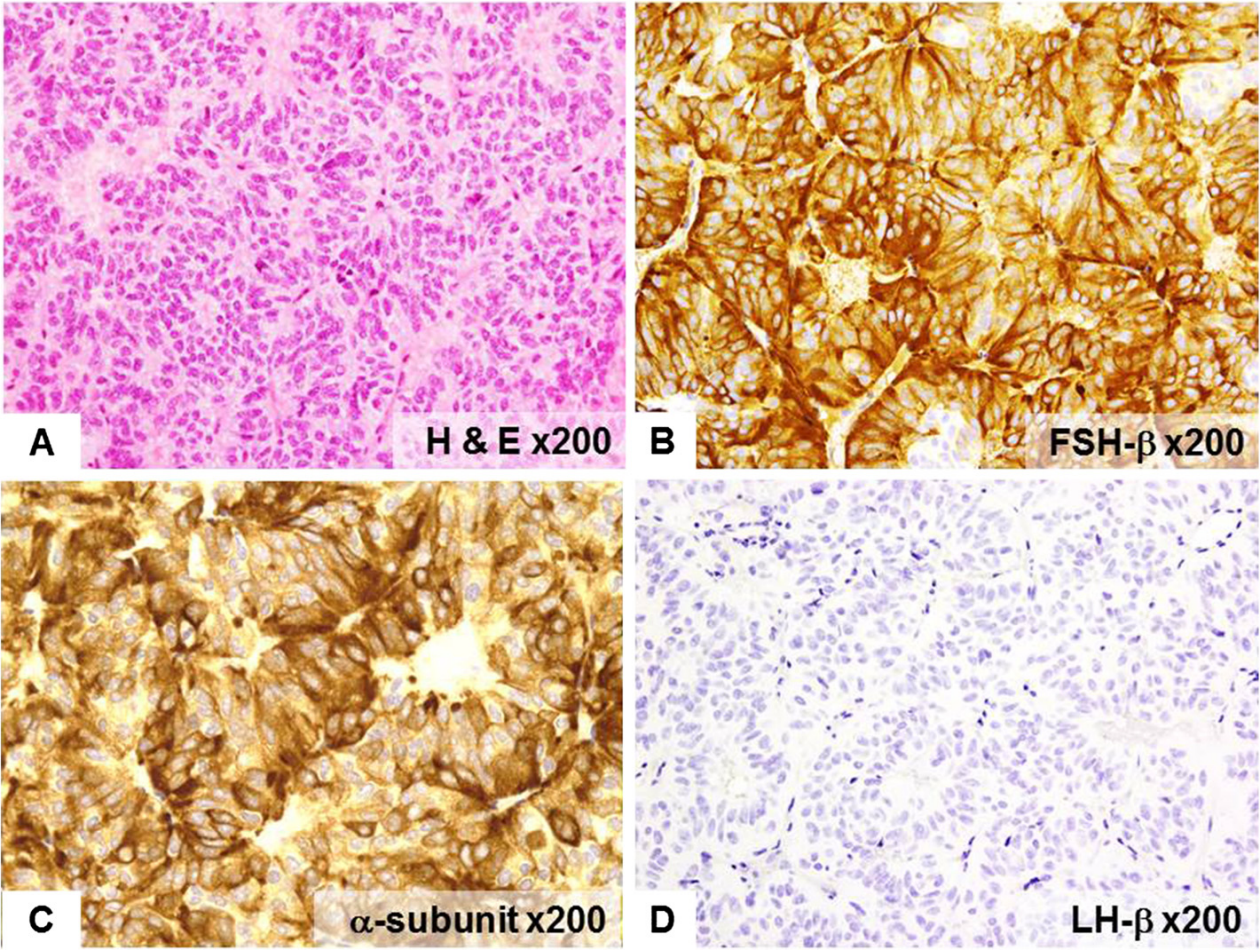
**Photomicrographs of the pituitary adenoma.** Hematoxylin-eosin staining showing acinar-like arrangement of the adenoma cells (**A**, ×200). Immunostaining revealing intense and diffuse staining for follicle-stimulating hormone-b (**B**: ×200) and diffuse staining for a-subunit (**C**, ×200), but no positive reaction to luteinizing hormone-b (**D**: ×200).

**Figure 4:**
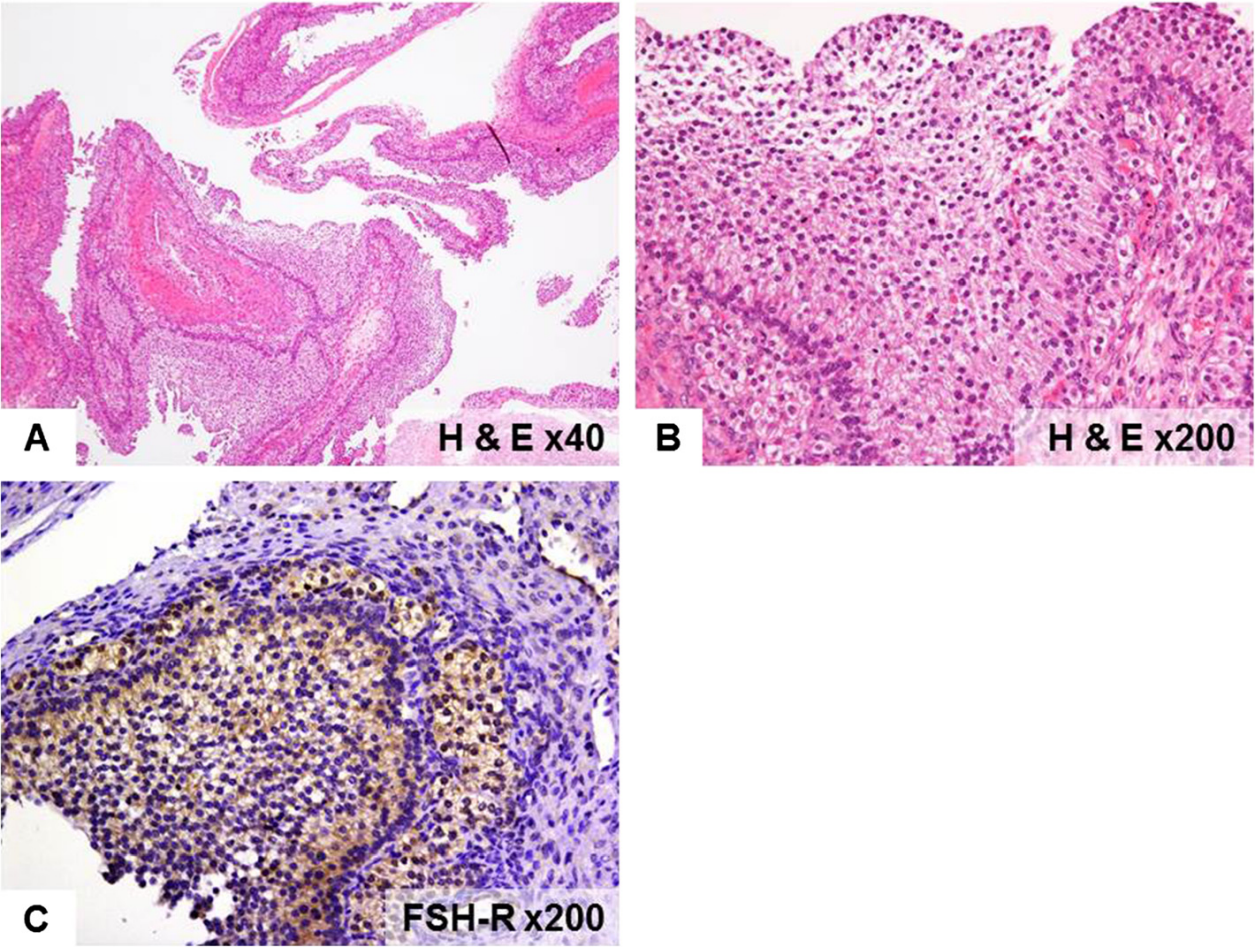
**Photomicrographs of the ovarian cysts.** Hematoxylin-eosin staining showing multiple enlarged follicular cysts (**A**, ×40) and stratified thick follicular cell layer (**B**, ×200). Immunostaining revealing intense staining of the follicular cells for follicle-stimulating hormone receptor (**C**, ×200).

Histological examination of the pituitary tumor demonstrated basophilic cells with hyperchromatic nuclei without atypism. These adenoma cells had acinar-like arrangement (Figure 3A). Immunohistochemistry showed positive reaction to anti-FSH-b antibody (1:200 dilution, Leica, Wetzlar, Germany) and a-subunit (Roche, Basel, Switzerland), but negative reaction to all other pituitary hormones including LH-b (1:400 dilution, Leica) (Figure 3B,C,D). The histological diagnosis was gonadotroph cell adenoma. Additional examination of the former ovarian cyst showed multiple large follicular cysts with stratified follicular cell layer (Figure 4A,B). This region showed positive immunohistochemical reaction to FSH-receptor staining (1:800 dilution, Abcam, Cambridge, UK) (Figure 4C).

## Discussion

A few cases of FSH-secreting gonadotroph cell adenoma manifesting as ovarian hyperstimulation have been reported with the endocrinological characteristics, but the true incidence of this condition including unnoticed or subclinical cases remains unclear [1,7-11]. We retrospectively reviewed patients who underwent surgical treatment for gonadotroph cell adenoma at Kohnan Hospital between April 2005 and March 2012. During this period, 200 patients were treated for gonadotroph cell adenoma including 26 women under the age of 50 years. Two (7.7%) of these 26 patients had a history of ovarian cysts and presented with normal to high FSH levels and suppressed LH level. Regretfully one patient’s follow-up data was missed in out patient department.

Age is the most fundamental characteristic for the diagnosis of this entity, because the response of the ovary to FSH stimuli is important for the onset of ovarian hyperstimulation. In postmenopausal women, FSH oversecretion cannot be recognized unless a very low LH level is noticed [12], so that all reported patients with ovarian hyperstimulation are of reproductive age. Although several FSH receptor mutations have been identified for very rare cases of spontaneous ovarian hyperstimulation [13,14], no pathological abnormalities have been identified in the ovary in most cases of ovarian hyperstimulation caused by FSH-secreting gonadotroph cell adenoma. Therefore, regression of the ovarian hyperstimulation can be achieved by resolving the inappropriate FSH secretion in such cases.

Endocrinological pattern is also an important characteristic in the diagnosis of this entity. Serum estradiol level is elevated in response to FSH stimulation, which is secreted in adenoma cells. Subsequently, the hypothalamus-anterior pituitary gland axis is suppressed due to the negative feedback mechanism. As a result, the excessive FSH level is reduced to the normal range. Serum LH level is also significantly reduced to below the lower limit of the normal range, probably due to the negative feedback mechanism or compression of the normal pituitary gland by the tumor [4,5,7]. Apparent normal FSH, suppressed LH, and high estradiol levels can be considered as the characteristic endocrinological profile of this FSH-secreting gonadotroph cell adenoma. Serum prolactin concentration was elevated in most cases, probably due to pituitary stalk compression by the sellar tumor, the so-called stalk effect [15]. This observation is considered to be a non-specific finding with large pituitary adenomas.

Ovarian hyperstimulation syndrome is recognized as a serious iatrogenic complication of fertility treatment using human menopausal gonadotropin combined with human chorionic gonadotropin [16,17] which also manifests as enlarged multicystic ovaries [18]. However, there are several differences between iatrogenic ovarian hyperstimulation syndrome and ovarian hyperstimulation caused by FSH-secreting gonadotroph cell adenoma. The most significant clinical difference is the change of vascular permeability. Iatrogenic ovarian hyperstimulation syndrome is generally characterized by a systemic increase of vascular permeability, manifesting as extravasation of fluid into the peritoneal cavity or third space, which does not occur in pituitary adenoma-induced ovarian hyperstimulation [6]. The correct diagnosis is difficult to establish based only on the ovary symptoms without systemic changes, because these patients are usually treated at the gynecological clinic without referral to the neurosurgical department. The present case had multiple ovarian cysts but no ascites.

The clinical severity of ovarian hyperstimulation varies greatly, because the biological activity of FSH is not always uniform. Our Case 2 was diagnosed with ovarian cysts and subsequently presented with visual disturbance. She was treated by adenoma surgery for the purpose of optic nerve decompression. However, retrospective review of her preoperative endocrinological examination suggested possible relationships with previous ovarian hyperstimulation, although several clinical data points were missing. Therefore, the gynecological history of patients with sellar tumor of reproductive age requires careful evaluation.

## Conclusion

The present case of FSH-secreting gonadotroph cell adenoma was the cause of recurrent ovarian cysts. We emphasize the importance of pituitary imaging and detailed endocrinological examinations as well as careful evaluation of the gynecological history in women of reproductive age to avoid unnecessary ovary surgery.

## Ethics

The therapeutic protocol was approved by the internal ethics committee of Kohnan Hospital 2012.

## Consent

Written informed consent was obtained from the patient for publication of this case report and accompanying images. A copy of the written consent is available for review by the Editor-in-Chief of this journal.

## Abbreviations

FSH: Follicle-stimulating hormone; LH: Luteinizing hormone.

## Competing interests

The authors declared that they have no competing interests.

## Authors’ contributions

TK analyzed the patient data regarding the endocrinological outcome, and was a major contributor in writing the manuscript. YO performed tumor removal all through the investigated period. KI had managed initial treatment for the disease. MW performed pathological examinations. And TT gave an essential suggestion and supervised this manuscript. All authors read and approved the final manuscript.
